# Present and Future of Prosthetic Dentistry

**Published:** 1893-01

**Authors:** Geo. H. Wilson


### Present and Future of Prosthetic Dentistry.

BY GEO. H. WILSON, D.D.S.

Read before the Ohio State Dental Society, December, 1892.

We can hardly consider Prosthesis of the present without a hasty resume of the past. I believe every thinking dentist has been impressed, when he has held in his hand a specimen of work made fifty or one hundred years ago, with the wonderful improvement of even the low-priced work of to-day. The specimens we see, of work carved from ivory of the elephant or hippopotamus, are meager excuses for lost dental organs. The very nature of the material precluded a proper textural appearance, except in rare instances. The method of construction, carving, precluded uniform pressure and absolute contact—the essentials of a suction denture.

Our venerable Dr. J. A. Robinson, of Jackson, Mich., quotes Dr. Flagg as saying, in 1836: "It was all we could expect, if the patient could answer questions in monosyllables without the plate coming down in the mouth." The same writer tells us that the first artificial teeth he saw were calves' teeth fastened to a leather base, worn for beauty, but not for use, as they were taken out while eating.

As this was in the twenties, it is very apparent that there was as great a contrast in workmanship and materials used as at the present day.

It was about 1820 that Dr. Plantou brought the first porcelain teeth from France. Though these were very crude, they certainly were more cleanly and nearer healthful than the calves'

and sheep's teeth, or those carved from ivory of the same period.

The porcelain teeth were either ligated to natural teeth, doweled to roots or riveted to metal bases. It was not until later that the composition of the porcelain was so improved that it would successfully withstand the soldering flame.

At the time of the introduction of vulcanite, in 1855, great improvements had been made in the composition and moulding of the porcelain, also in the adapting, and attaching to the bases, of gold and silver.

It was during the preceding eleven years that Dr. John Allen had been experimenting with and had partially introduced the most capable of all materials for Prosthesis, continuous gum. We all know how everything else was supplanted by the "boiled rubber," and we are acquainted with the woeful wail that has gone up from the good men of the profession over the vile stuff, as it is often called.

But, gentlemen, we have been mistaken; it has been one of the greatest blessings (in disguise) that the profession and public have ever known. Think of the multitude of poor people it has served with masticators; of the number of ladies, full of pride, but little wealth, who would have been deprived of a pleasing smile. So I say it has given health and comfort to many, and pleasure and delight to all.

I affirm, also, it has been a great blessing to the profession; it has done more than all else to show the contrast between the good and indifferent workman. It has brought the dentist in contact with a greater number of patients, giving him an opportunity to impress upon them the importance of saving the teeth and the unhealthfulness of the diseased tissues and organs. It has created a demand for dentistry as never before. One of the strongest arguments in favor of its usefulness is the fact that, notwithstanding the amount of abuse to which it has been submitted, it still lives.

The last great innovation is "Crown and Bridge Work." Though it is scarcely a decade since the present form of bridge-work was first placed before the profession, yet to-day it has so firmly established itself that it takes a very courageous man, if he happens to have a limited practice, to say, when his patient asks for it, that he does not do that kind of work. Then, with the recent graduate he supposes it is the *sine qua non* of success; that, possessing this knowledge, his position in the profession and society is assured, and he has but to enter in and possess. It is also the goal of the average student's ambition. He counts his time lost until he can get at work upon the "one thing in dentistry."

It seems to me that no observant man can doubt that this bridge work has sacrificed more good teeth, and worked far more injury to the health of the wearer, in the same length of time, than vulcanite. Notwithstanding all the abuse of vulcanite and the bridge, I believe that up to the present time they have had more to do with the upward progress of dentistry than any other two materials or processes.

Let us study for a few moments the influence the bridge has had upon the public and the profession. The public has been educated to desire something better and to pay for more than a five or ten dollar piece of vulcanite. This being conceded, there is no longer any excuse for a man's confining himself to vulcanite as the only base for artificial work.

The effect upon the dentist himself has been, or should have been, more marked than upon the patient. With the methods and skill required to work the precious metals, and his failures to stimulate him to more conscientious and painstaking service, he has developed a skill never reached before.

Not only has it taught him manipulation, but, being a new and unexplored field, it has created thought; it has brought to his mind more perfect contour, symmetry, and harmony, and the effects upon the health of the contiguous tissues.

I would not be understood as implying that this mental process has been confined to the advent of bridge work, because it has not. It has only stimulated it. We can trace it back to the beginning of modern dentistry. The human mind is won't to go to the extremes and gradually contract to the perfect form or idea.

Thus we see the contrast between animal's teeth as used for substitutes, and mineral teeth; between carved ivory and imperfectly swaged metal bases as compared with the moulded plastic, vegetable bases, and also between removable and cemented attachments, each successive step introducing more truth, though often hedged about by fallacies. So I believe we are on the threshold of an important era in Prosthesis, and that 1900 will see a wonderful development.

While this department of our profession has been considered degraded and degrading, I opine that the time will soon come when it will be considered that the prosthetic dentist requires more and finer manipulative skill, and more artistic perception and cultivation, and consequently will be better remunerated.

What are the factors that are working and will work for this development? I would first mention that there is room for improvement. Some one has said that the greatest room in this wide world is the room for improvement. Another reason is that the profession and public demand something better. Still another, and one that demands our attention, is the lengthening of the college courses. Under the old regime of two winters, a cramming process was implied, with insufficient time for manipulation. With half as much more time, greater time for possibilities is given. Still there is no time for letting up upon the part of either the faculty or student, but time is given for more and a wider range of work.

There seems to be a tendency in many of the colleges to devote a portion of the time, the first year at least, to technic work, so that the student, before he goes to work upon the mouth, is thoroughly familiar with the instruments, tools, materials, procedures and processes. Having acquired all this beforehand, when he comes to the practical case, he is prepared to comprehend the conditions and requirements, and make a study of the principles involved.

We observe that there is a disposition to still further lengthen the term. The Medical Department of the University of Michigan has been agitating a very radical change. They now have a course of four years, of nine months each, and talk of extending it to six years, of nine months each, and, at the same time confer the two degrees of B. S. and M. D. There is food for thought. As this growth in our specialty must come by education, there is one factor more I wish to mention—the Columbian Exposition. It is designed that this Exposition shall far surpass anything of its kind. It is to be not only an aggregation of the material products of men's minds and hands, but it is to be a congress of the men themselves. It is designed that the specialists shall be able to come in touch with the mind of the whole world, thereby gaining the spirit of enthusiasm that has animated these noble minds. We are not only to absorb the spirit, but we are to compare ourselves. What, if we are humiliated by the comparison — we, who have boasted so much of American dentistry?

